# An Update on Animal Models for Severe Acute Respiratory Syndrome Coronavirus 2 Infection and Countermeasure Development

**DOI:** 10.3389/fmicb.2021.770935

**Published:** 2021-11-08

**Authors:** Liang Zhang, Shuaiyin Chen, Weiguo Zhang, Haiyan Yang, Yuefei Jin, Guangcai Duan

**Affiliations:** ^1^Department of Epidemiology, College of Public Health, Zhengzhou University, Zhengzhou, China; ^2^Suzhou Institute of Systems Medicine, Chinese Academy of Medical Sciences, Suzhou, China; ^3^Henan Key Laboratory of Molecular Medicine, Zhengzhou University, Zhengzhou, China

**Keywords:** COVID-19, SARS-CoV-2, ACE2, infection, animal model

## Abstract

Coronavirus Disease 2019 (COVID-19) caused by severe acute respiratory syndrome coronavirus 2 (SARS-CoV-2) has become a pandemic since March 2020 and led to significant challenges to over 200 countries and regions all over the world. The establishment of highly pathogenic coronavirus animal model is beneficial for the study of vaccines and pathogenic mechanism of the virus. Laboratory mice, Syrian hamsters, Non-human primates and Ferrets have been used to establish animal models of emerging coronavirus infection. Different animal models can reproduce clinical infection symptoms at different levels. Appropriate animal models are of great significance for the pathogenesis of COVID-19 and the research progress related to vaccines. This review aims to introduce the current progress about experimental animal models for SARS-CoV-2, and collectively generalize critical aspects of disease manifestation in humans and increase their usefulness in research into COVID-19 pathogenesis and developing new preventions and treatments.

## Introduction

Severe acute respiratory syndrome coronavirus 2 (SARS-CoV-2) is an enveloped positive-strand RNA virus, a member of the β-coronavirus genus, whose envelope spike protein binds to the cell-surface specific receptor angiotensin-converting enzyme 2 (ACE2) to initiate the infection (Zhou P. et al., 2020). SARS-CoV-2 is a recently emerged human pathogen and causes a highly contagious transmittable disease named Coronavirus Disease 2019 (COVID-19). Globally, 1 October 2021, there have been 233,503,524 confirmed infections of COVID-19, including 4,777,503 deaths, reported by the World Health Organization (WHO) (WHO, 2021). The main symptoms of patients are weakness, dry cough, fever, severe dyspnea, and even death (Fu et al., 2020). After the outbreak, researchers across the world rapidly reported a series of studies on SARS-CoV-2 and COVID-19, including the SARS-CoV-2 genome sequence (Harcourt et al., 2020; Zhu et al., 2020), the clinical symptoms of infected patients (Chen et al., 2020; Huang et al., 2020), the corresponding treatment strategies (Jin et al., 2020), and the development of vaccines (Gao et al., 2020; Wang et al., 2020). Nowadays, many clinical trials about vaccine effectiveness are under way around the world, supported by the development of animal infection models. Preclinical studies on different model organisms will not only help to understand the basic characteristics of SARS-CoV-2 virus and its pathogenesis, but also help to better understand and test the safety and effectiveness of therapeutic drugs and vaccines. This review summarized the characteristics of various animal models of SARS-CoV-2 since the outbreak, and analyzed the application scope of different experimental animals (Table 1), so as to provide certain theoretical references for optimizing and selecting animal models of COVID-19 in the future.

**TABLE 1:** Comparison of features of experimental animals susceptible to SARS-CoV-2.

Features	Animals
**Route of challenge**	
Intranasal	All animal except for Asia lion reported above
Oral intraocular	Rhesus monkey, Cynomolgus macaques
Ocular	Hamsters, Rhesus monkey, tree shrew

**Virus detection**	
Upper respiratory	hACE2 mice, hamsters, ferrets, cats, tree shrew and all of the above NHPs
Lower respiratory	hACE2 mice, hamsters, ferrets and all of the above NHPs
Feces/Fecal swab	Aged hACE2 mice (30-week-old), hamsters, cats and ferrets, rhesus monkey, AGM
Throat swab	Rhesus monkey, ferrets, rhesus monkey, AGM

**Symptoms**	
Fever	Ferrets, rhesus monkey, cynomolgus macaques,
Weight loss	hACE2 mice, hamsters, rhesus monkey
Shortness of breath	Hamsters, rhesus monkey, AGM

**Airborne transmission**	Hamsters, ferrets, Cat

**Severity of pneumonia**	
Simulate asymptomatic carriers	Tree shrew, dog, callithrix jacchus, cynomolgus macaques (25%)
Simulation of mild symptoms	hACE2 mice, ferrets, cat, cynomolgus macaques and rhesus monkey, young AGM (all male and all ∼3.5 years of age)
Simulation of severe symptoms	Hamsters, aged AGM (13–15 years of age/ARDS)

*NHPs, Non-human primates; AGM, African green monkey; ARDS, acute respiratory distress syndrome.*

## Mice Models

The laboratory mice are the most common animal used for models of infectious diseases due to their advantages of high adaptation to different environments, low cost, and physiological similarities with humans (Brehm et al., 2010; Walsh et al., 2017). Actually, C57BL/6 and BALB/c mice are not susceptible to SARS-CoV-2, since the main barrier of the infection of mouse (Mus musculus) cells with SARS-CoV-2 is the lack of appropriate receptors for viral entry. It’s known that SARS-CoV-2 utilizes the cellular surface receptor ACE2 to enter host cells, while mouse ACE2 can’t effectively bind the viral spike protein. Currently, several strategies have been developed to solve it.

### Non-transgenic Mouse

At present, the main research progress on the mouse model of SARS-CoV-2 infection focuses on the following aspects: (a) Obtain mouse adapted SARS-CoV-2 strain through domestication; (b) Adenovirus (Ad5) or adeno-associated virus (AAV) mediated expression of human ACE2 (hACE2).

Through the adaptive screening of clinical isolates of SARS-CoV-2 by inoculating the virus into mouse specific tissues, the virus can adapt to its specific living environment, and mutate under specific selection pressure, and finally increase the infectivity, so as to achieve the purpose of more conducive to its effective replication. As previously reported, the mice model was induced by intranasal inoculation with clinical isolates of SARS-CoV-2, by continuous passage in the lungs of elderly mice, to get the adaption of SARS-CoV-2 in mice (Gu et al., 2020). Finally, the 6th generation of the mouse adapted strain (MASCP6) increased infectivity in the lungs of mice, resulting in interstitial pneumonia and inflammatory response in both young and elderly mice after intranasal inoculation. MASCP6 infection caused interstitial pneumonia at 3 days after inoculation, and the lung injury at 5 days after inoculation was milder than that at 3 days after inoculation, suggesting a process of self-recovery. The MASCP6 genome contains five mutations in comparison with its original strain, and these mutations resulted in the changes of four amino acid residues in the ORF1ab, S, and N proteins, respectively. The structural reconstruction showed that the substitution of N501Y in the receptor binding domain of S protein increased the binding affinity of S protein to ACE2 in mice. The A23063T mutation led to the substitution of N501Y amino acid in the receptor binding domain of S protein, and the proportion of A23063T mutation gradually increased after the first passage in the mouse lung homogenate. Therefore, the increased virulence of MASCP6 in mice may be due to the rapid occurrence of N501Y in the S protein receptor-binding domain. Additionally, whether three other mutations in addition to N501Y also modulate viral infection remains to be determined. It was worth noting that no visible clinical symptoms and weight loss was recorded throughout the experiment with a relative low dose of MASCP6 challenge. Therefore, it remains to confirm whether a higher dose of MASCP6 will aggravate the lung pathology.

Ad5 or AAV vectors have been widely used in a variety of animal models due to their advantages of wide host-range, fast proliferation rate and no insertion mutation. In the study of COVID-19, Ad5-hACE2-sensitized mice or AAV-hACE2-sensitized mice were used to investigate the pathogenic mechanism of SARS-CoV-2 because of their advantages of fast modeling time and avoiding the influence of traditional gene modification techniques. It had been reported that replication-defective adenoviruses encoding hACE2 were introduced into BALB/c mice via intranasal administration leading to receptor expression in lung tissues. Further experiments found that hACE2-transduced mice were productively infected with SARS-CoV-2, and high lung viral titers, lung pathology, and weight loss were observed (Hassan et al., 2020). Mice sensitized by AAV-hACE2 vector were also susceptible to SARS-CoV-2 infection, but virus replication appeared to be lower than that of mice sensitized by Ad5-hACE2. Notably, delivery of hACE2 by adenovirus transfection infected both normal and immunodeficient mice with SARS-CoV-2 (Sun J. et al., 2020). The virus replicated briefly in the lungs for only a few days, and the immune system could remove the virus in about 7 days. Ad5-hACE2-sensitized mice also contributed to the evaluation of SARS-CoV-2 specific therapy, for example, the transfer of plasma from patients recovering from infection or remdesivir (Sun J. et al., 2020). The results from Ad5-hACE2-sensitized mice indicated that either convalescent plasma from COVID-19 patients or remdesivir could protect mice from SARS-CoV-2 infection (Pruijssers et al., 2020). Above therapies alleviated weight loss, and significantly accelerated viral clearance and reduced inflammatory cell infiltration in lung tissues of mice (Grein et al., 2020). Although Ad5-transduced mice are of broad and immediate utility to explore COVID-19 pathogenesis and to assess novel therapies and vaccines, the model still has some limitations. First of all, Ad5-hACE2-transduced mice did not develop into severity after SARS-CoV-2 infection (Grein et al., 2020). Secondly, the mice also did not develop extrapulmonary manifestations of the disease. However, research into the pathogenesis, particularly of acute respiratory distress syndrome (ARDS), will require the development of infection models that include mild and severe illness.

### Golden Syrian Hamster

The golden Syrian hamster had been reported in previous studies to be a widely used animal model that can support the replication of SARS-coronavirus (SARS-CoV) (Sims et al., 2005; Roberts et al., 2008). The SARS-CoV-2 isolates were also able to replicate effectively and caused severe pathological damage in the lungs of Syrian hamsters (Chan et al., 2020), similar to the radiographic features commonly reported in patients with COVID-19 pneumonia.

SARS-CoV-2 strains isolated from nasopharyngeal aspirates and swabs of confirmed COVID-19 patients were cultured in Vero E6 cells and inoculated intranasally in hamsters. It had been reported that conjunctivitis was observed in COVID-19 patients (Lu C.W. et al., 2020). As expected, in addition to viral infection by the nasal route, there was also infection by the intraocular route (Imai et al., 2020). After infection, hamsters showed rapid breathing, weight loss, alveolar damage, and extensive cell apoptosis (Takayama, 2020), which was similar to the development of mild to moderate disease in humans (Struyf et al., 2020). It was worth noting that previous study had shown that virus replication was higher in the upper respiratory tract (turbinate) than in the lower respiratory tract (lung) (Chan et al., 2020). In hamsters infected with SARS-CoV, the viral load in the turbinate was generally similar or lower than that in the lungs (Roberts et al., 2005). These lines of evidence suggest that SARS-CoV-2 infects the upper respiratory tract no less than the lower respiratory tract, which may explain why SARS-CoV-2 is more infectious. However, subsequent studies (Imai et al., 2020) observed that the virus replicated efficiently in the respiratory tracts of both young and older infected animals, with no difference in growth in the upper and lower respiratory tracts. This is worthy of further study. Compared with other mice, golden hamster was more sensitive to SARS-CoV-2, which was suitable for the study of COVID-19. Additionally, because the aerosol pathway can effectively transmit SARS-CoV-2 to immature hamsters (Imai et al., 2020), golden hamster is also of great significance to study the propagation kinetics of COVID-19. Taken together, this cheap and convenient hamster model is an important tool for studying the transmission, pathogenesis, treatment, and vaccine evaluation of SARS-CoV-2.

### Transgenic Mice

As mentioned above, mice have been widely used in various studies due to their clear genetic background. However, numerous studies found that wild-type mice (C57BL/6 or BALB/c, etc.) were not susceptible to SARS-CoV-2, and mouse ACE2 (mACE2) could not bind to COVID-19 virus in its natural state (Bao et al., 2020; Dinnon et al., 2020). It was known that hACE2 was a functional receptor of SARS-CoV-2 (Zhou P. et al., 2020). Genetically modified hACE2 mice were demonstrated to be susceptible to SARS-CoV-2. Therefore, a variety of hACE2 mouse models were established on this basis to simulate and explore the pathogenesis and disease recovery after SARS-CoV-2 infection. According to different mouse construction strategies and methods, the current SARS-CoV-2 infected transgenic mouse models can be divided into the following two types: (a) Human host cell SARS virus binding receptors, such as ACE2, were introduced into mice; (b) The related genes were deleted by gene editing technique in mice.

At the earliest, researchers constructed the expression vector of hACE2 gene initiated by mouse ACE2 promoter, and then obtained the hACE2 transgenic mice by microscopic injection. SARS-CoV-2 strain was inoculated nasally into hACE2 transgenic mice to observe the response of viral infection. The results showed that SARS-CoV-2 did not infect wild-type mice, but was susceptible to infect hACE2 transgenic mice. After SARS-CoV-2 was inoculated into hACE2 transgenic mice, significant weight loss and interstitial pneumonia were observed in hACE2 mice, and macrophages were observed to cluster in the alveolar lumen, and a large number of macrophages and lymphocytes were infiltrated into the alveolar stroma (Bao et al., 2020). In addition, it was showed that first infected mice were resistant to high doses of SARS-CoV-2 once they were re-infected (Jiang et al., 2020). It was also reported that SARS-CoV-2 infection successfully induced pneumonia in hACE2 mice, and the pathological features in lung tissues were extremely similar to those of severe patients (Xu Z. et al., 2020).

With the rapid development of CRISPR/Cas9 genome editing technology in recent years, animal models suitable for the study of COVID-19 can be constructed simply by genetic modification of mice. CRISPR/Cas9 knock in technology was used to insert complete hACE2 cDNA into mACE2 gene, resulting in gene insertion inactivation. After that, mACE2 gene could not be expressed normally, while the inserted hACE2 gene was expressed under the regulation of the promoter of mACE2 gene. The hACE2 gene contained IRES site, and tdTomato gene was also inserted into the downstream of hACE2 gene to achieve co-expression, so as to improve mRNA stability and translation efficiency. Therefore, hACE2 transgenic mouse was successfully constructed by injecting Cas9 mRNA, sgRNA and targeted constructs into the fertilized eggs of mice. Compared with wild-type C57BL/6 mice, young and old hACE2 transgenic mice that were nasally infected with SARS-CoV-2 maintained high viral loads in the lungs, trachea, and brain (Sun S.H. et al., 2020). Although no deaths were observed, interstitial pneumonia and elevated cytokines were detected in transgenic mice infected with SARS-CoV-2 (Bao et al., 2020). In addition, the results also showed that intragastric injection of SARS-CoV-2 might also cause infection and lung damage in humanized ACE2 mice.

Taken together, the hACE2 transgenic mice were susceptible to SARS-CoV-2 to different degrees, and infected animals showed the clinical symptoms such as weight loss and lung disease observed in human infections.

## Non-Human Primate

Non-human primates are genetically close to humans and possess similar anatomical structures (Colman, 2018). As such, it is often the gold standard for preclinical trials and the final step in the preclinical development of drugs and vaccines. In previous studies on respiratory viruses such as SARS-CoV and Middle East respiratory syndrome coronavirus (MERS-CoV), Non-human primates showed susceptibility. In the study of SARS-CoV-2, Non-human primates also showed susceptibility. Rhesus monkeys, crab monkeys and marmosets had been used as animal infection models of SARS-CoV-2 and simulated clinical symptoms of human infection, including fever, diarrhea and pneumonia, to a certain extent (Le Bras, 2020; Lu S. et al., 2020).

### Rhesus Monkey

Rhesus monkeys could be infected with SARS-CoV-2 through intranasal, intratracheal, oral, and binocular inoculations (Rockx et al., 2020; Shan et al., 2020). The gastrointestinal tract had also been documented as an alternative route of SARS-CoV-2 infection in non-human primate models (Jiao et al., 2021). The infected rhesus monkeys developed fever, irregular breathing, loss of appetite, weight loss, pallor, and dehydration. High virus content was detected in nasal, pharyngeal and bronchoalveolar lavage fluid, and pulmonary Computed Tomography (CT) showed obvious osmotic characteristics (Shan et al., 2020). The lungs of most rhesus monkeys presented with pulmonary infiltration, edema, and alveolar congestion, and infected animals reflected the clinical symptoms and respiratory pathology of moderate COVID-19. Clinical symptoms of weight loss and frailties were found in both young and old rhesus monkeys. The viral load was the highest at 3 days after respiratory tract infection in young and old animals. However, viral replication in nasopharyngeal, anal and lung swabs was significantly higher in older rhesus monkeys than that in younger rhesus monkeys, and viral antigens were detected in alveolar epithelial cells and macrophages (Yu P. et al., 2020). Infected rhesus monkeys presented with typical interstitial pneumonia with thickened alveolar septum, inflammation, and edema. In particular, diffuse severe interstitial pneumonia was found in elderly rhesus monkeys, which was similar to the pathologic features of human lungs (Mohanty et al., 2020). It can be speculated that the severity of lung disease after SARS-CoV-2 infection may be related to the age of the animal, which is consistent with the age-related characteristics of SARS-CoV-2 infection in humans. It had been reported that old age was a risk factor for severe illness and death in human infections (Dudley and Lee, 2020; Yang et al., 2020; Zhang et al., 2020).

At present, it is unclear whether patients with confirmed COVID-19 will be reinfected after recovery. In addition to the study of primary infection, a rhesus macaque model of SARS-CoV-2 re-infection was also established to explore the risk of re-infection in recovered patients. Recovered rhesus monkeys were found to be less susceptible to re-infection when re-exposed to SARS-CoV-2 (Chandrashekar et al., 2020; Deng et al., 2020). During the early recovery phase of the initial SARS-CoV-2 infection, rhesus monkeys re-infected with the same SARS-CoV-2 virus strain showed no detectable viral clinical or histopathological findings. Compared with primary infection, the cellular immunity of re-infection showed a significant increase in antibody and immune response (Deng et al., 2020). It is speculated that SARS-CoV-2 elicits a rapid immune response in rhesus monkey after the first attack, thereby protecting the host from re-infection. Severe exposure to SARS-CoV-2 prevented re-infection in rhesus monkeys, which was confirmed in another study (Chandrashekar et al., 2020). But this situation should not be overexplained, as other studies had shown that the titers of neutralizing antibodies were so low as to be undetectable in previously infected patients. The immunological relevance of infection is still unclear, and more research is needed to demonstrate the durability of natural immunity. On this basis, rhesus monkey is helpful to evaluate the efficacy of candidate vaccines and to formulate treatment plans.

It was found that different virus strains and virus infection routes might affect lung diseases through the existing animal model of rhesus monkey, which provided some support for further study of the pathogenesis of the virus. However, rhesus monkey model reported to date have shown only mild to moderate clinical symptoms after SARS-CoV-2 infection, and no severe or even death has been observed (Cao et al., 2020). Therefore, further research is needed to establish and develop rhesus monkey model with severe clinical manifestations of COVID-19.

### Cynomolgus Macaques and Callithrix Jacchus

The infection in cynomolgus macaques and Callithrix jacchus differs little from that in rhesus monkeys, where SARS-CoV-2 is usually inoculated into the nasal cavity, mouth or conjunctival (Finch et al., 2020; Rockx et al., 2020; Ishigaki et al., 2021). Cynomolgus macaques infected with SARS-CoV-2 showed significant increase in body temperature, loss of appetite, and weight loss. No cynomolgus macaques showed clinical scores that reached a humane endpoint, therefore, cynomolgus macaques showed mild clinical symptoms. Viral pneumonia and/or bronchopneumonia have been observed in cynomolgus monkeys infected with SARS-CoV-2. Chest radiographs showed ground glass in the lungs, especially in peripheral areas, and SARS-CoV-2 was detected mainly in the nose and mouth of cynomolgus macaques for 1 week (Ishigaki et al., 2021).

SARS-CoV-2 spread in the respiratory tissue of cynomolgus macaques and caused clinical symptoms, suggesting that the crab-eating macaque model may be useful in evaluating vaccination and treatment. In addition, some cynomolgus macaques’ virus excretion was similar to human asymptomatic infection, the virus existed in the upper respiratory tract for a long time, and the viral RNA peak was reached at the early stage of infection. Delayed shedding and low antibody response of the virus in this model might explain the prolonged shedding of the virus and re-infection in human patients (Shi D. et al., 2020). The marmosets infected with SARS-CoV-2 showed no significant increase in body temperature, and only a few showed a slight increase (about 0.5°C) (Rockx et al., 2020). There was slight lung injury when the pathological section of lung tissue was observed. No virus was detected in any tissues of either Jacchus (Lu S. et al., 2020).

Collectively, Rhesus macaques are most sensitive to SARS-CoV-2 infection, which is similar to human infections with virus tissue distribution, lung changes and changes in cytokine and antibody levels, followed by cynomolgus macaques. Callithrix jacchus is least sensitive to SARS-CoV-2 infection (Lu S. et al., 2020). In fact, Rhesus macaque models were more widely used in the evaluation of SARS-CoV-2 vaccines (van Doremalen et al., 2020; Yu J. et al., 2020).

### African Green Monkey

African green monkey (AGM) is an ancient non-human primate species and the natural host of Simian immunodeficiency virus (Pandrea et al., 2006). In a series of studies on the physiological and pathological mechanisms of SARS-CoV-2 infection in young male AGM by aerosol or mucosal exposure, COVID-19 disease in young adult male AGM was found to be mild (Hartman et al., 2020), reflecting what often happens in healthy young adults infected with SARS-CoV-2 (Kim G.U. et al., 2020). Infection led to a low-grade fever in young AGMs, and respiratory symptoms were limited to a transient reduction in tidal volume. It was worth noting that all infected animals shed the virus from both respiratory and gastrointestinal tracts (Kim G.U. et al., 2020). Viral RNA was found throughout the respiratory and gastrointestinal systems, with higher levels found in gastrointestinal tissues (Kim G.U. et al., 2020). Young AGM infection model well simulated the status of mild/subclinical COVID-19 disease in humans and provided insight into live virus shedding (Hartman et al., 2020).

But except for mild clinical conditions, SARS-CoV-2 also caused a range of serious illnesses, from asymptomatic infections to life-threatening illnesses, especially in the elderly AGM. ARDS is a common and often fatal condition in COVID-19 patients. Previous studies found that non-human primates were susceptible to SARS-CoV-2 infection and progressed to mild-moderate disease, but none had been able to display severe disease or clinical rapid deterioration in patients with ARDS. Recently, two cases of ARDS in aged AGMs infected with SARS-CoV-2 have been reported (Blair et al., 2021). The pathological lesions and disease in these monkeys were similar to severe human infections. With the challenge of SARS-CoV-2 via two routes (aerosol and multi-routes) in AGM, older AGM developed into ARDS and a significant increase in circulating cytokines was detected. The increase of circulating cytokines was a common phenomenon in severe patients with COVID-19. Previous studies suggest that ARDS is often associated with an increase in circulating inflammatory cytokines, a condition known as cytokine storms (Mangalmurti and Hunter, 2020; Hu et al., 2021). In addition, the plasma level of interleukin (IL)-6, a predictive marker and putative treatment target in SARS-CoV-2 infections, was elevated in infected AGMs. This situation was also shown in another study (Woolsey et al., 2021). Additionally, infected AGM could prevent re-infection after being challenged again within 35 days of exposure (Woolsey et al., 2021). It is speculated that the AGM infection models can be used to analyze the host immune response and study the pathogenesis of COVID-19. From the above studies, we conclude that AGM has the ability to model SARS-CoV-2 infection and elderly AGM is helpful for modeling severe disease manifestations.

## Mammal Models

### Ferret

The anatomical proportions of the upper and lower respiratory tracts and the density of submucosal glands in the bronchial wall of ferrets are very similar to the condition of the human respiratory tract. Therefore, ferrets are often used as experimental animal models to study respiratory viruses. In previous studies on SARS-CoV, ferret infection models had been used to evaluate the effectiveness of potential therapeutic effects for SARS (Chu et al., 2008; See et al., 2008; Park et al., 2020), and ferret ACE2 had also been proven to contain key SARS-CoV-binding residues (Wan et al., 2020). Recently, ferret has been selected as the model for SARS-CoV-2 study (Kim Y.I. et al., 2020; Liu et al., 2020; Shi J. et al., 2020). SARS-CoV-2 strain was used to infect ferrets through the nasal route. The physical condition of the ferrets after infection was characterized by a slight increase in body temperature, but no other clinical symptoms, and no serious illness or death. Viral RNA was detected in all ferrets’ turbinate, soft palates and tonsils. SARS-CoV-2 was detected in the upper respiratory tract of all ferrets directly infected for the first time, indicating rapid SARS-CoV-2 infection, consistent with previous human case reports (Guan et al., 2020). Importantly, SARS-CoV-2 was found in nasopharyngeal swabs from all ferrets in direct contact with infected ferrets, as well as from ferrets in adjacent cages, suggesting airborne transmission (Kim Y.I. et al., 2020). Current studies suggest that ferret animal models are naturally susceptible to SARS-CoV-2 (Guan et al., 2020; Kim Y.I. et al., 2020). The infection caused mild clinical symptoms similar to human infections. However, there are still some limitations. This animal model has mild clinical symptoms, no weight loss or even death, and low pulmonary virus titers. It is difficult to study the clinical characteristics of severe and critical patients with COVID-19. Due to the high susceptibility of ferrets, this infection model provides a useful tool for studying the transmission of SARS-CoV-2.

### Tree Shrew

Tree shrews, a class of mammals, had been shown to be close to primates in previous studies (Fan et al., 2013, 2019). Therefore, due to its unique characteristics, it is being developed as an emerging experimental animal that may replace primates in biomedical research. In fact, in previous studies, tree shrews had been used in animal models of several viral infections, including, influenza virus (Li et al., 2018), Zika virus (Zhang et al., 2019), etc. Therefore, numerous studies have used tree shrew as an animal infection model to test whether it is susceptible to SARS-CoV-2.

For tree shrew infection experiments, the SARS-CoV-2 strain was usually inoculated by oral, intranasal and intraocular routes (Xu L. et al., 2020; Zhao et al., 2020; Kayesh et al., 2021). After infection, viral RNA was detected in different organs and tissues (e. g. conjunctiva, kidney, bladder, small intestine, testis and ovary) of adult tree shrews, and the peak of viral RNA in lung tissues appeared at day 3 post-infection. Compared with adult tree shrews, viral RNA was only detected in a small number of lung lobes from elderly animals, and the peak of viral RNA appeared at day 7 post-infection. Above results suggest that older tree shrews seem to be less likely to be infected with SARS-CoV-2 than adult tree shrews. It is a sharp departure from previous reports that older people are more susceptible than young individuals (Zhou Z. et al., 2020). Therefore, this observation needs to be studied further. Taken together, above results indicate that tree shrews are sensitive to SARS-CoV-2 infection. However, another previous study observed that tree shrews inoculated with SARS-CoV-2 showed no clinical symptoms except increased body temperature, especially in female animals (Zhao et al., 2020). The histopathological examination of the young animals showed that lung abnormalities were the main changes, while other tissues displayed slight changes. Additionally, the virus exudation and replication levels were relatively low in tree shrews (6–12 months old), adult tree shrews (2–4 years old) and aged tree shrews (5–7 years old). According to the comparison of the two studies (Zhao et al., 2020; Kayesh et al., 2021), the different concentrations of injected virus and the different routes of infection led to above differences, which need further study to clarify. Notably, young tree shrews did not experience a significant increase in body temperature for 6 days after inoculation, but virus shedding was detected from the nose, throat, anus and serum. Therefore, tree shrews were asymptomatic after SARS-CoV-2 infection at young age, suggesting tree shrews might be an intermediate host of SARS-CoV-2 or asymptomatic carriers.

### Cat and Dog

Previous studies showed that domestic cats were susceptible to the SARS-CoV and that they could effectively transmit the virus to previously uninfected animals (Martina et al., 2003). Based on the three-dimensional molecular structure prediction model of viral S protein and ACE2 protein complex, SARS-CoV-2 was predicted to identify ACE2 in other animals such as cats, whose ACE2 protein is similar to the key residues of the bound virus (Gaudreault et al., 2020; Wan et al., 2020). By intranasally inoculation, viral RNA was detected in the oracle bone, soft palate, tonsil, lung, and small intestine of domestic cats at day 3 post-infection, suggesting that SARS-CoV-2 can also replicated effectively in cats. Histopathology of cats at day 3 and 6 after infection revealed extensive lesions in the mucosal epithelium of the nose and trachea, and in the lungs. Like ferrets, infected cats showed no obvious clinical symptoms. Notably, SARS-CoV-2 infection was observed in non-inoculated cats in cages with infected cats, suggesting that cats are susceptible to contact transmission route (Halfmann et al., 2020). Fortunately, domestic cats infected with high doses of the virus in the laboratory can only transmit SARS-CoV-2 to other cats, but there is no evidence that domestic cats can infect humans.

Previous studies found that dogs were not susceptible to SARS-CoV-2. Viral titers were performed on oropharyngeal and rectal swabs from dogs and viral RNA was not detected in organs and tissues from infected dogs. In addition, infectious virus was also not detected in any swab taken from these dogs. These results suggest that dogs have a low susceptibility to SARS-CoV-2.

### Asiatic Lions

In May 2021, the SARS-CoV-2 strain was found in an Asian lion in a zoo in India. In the early stage of infection, some wild lions showed signs of loss of appetite, runny nose and occasional cough. Two of the infected lions died of COVID-19. After sequence analysis and phylogenetic analysis, the researchers concluded that SARS-CoV-2 is a variant strain of B.1.617.2 (Delta) (Mishra et al., 2021). As the park manager strictly abided by the COVID-19 guidelines and did not introduce any new animals to the zoo during the widespread COVID-19 pandemic in India, it is reasonable to suspect that the main source of the lion’s infection with SARS-CoV-2 may be asymptomatic. Lions share a common habitat, shelter, foraging space and water source, providing opportunities for close contact. Two types of lions exhibited in different enclosures sharing the same moat were also infected. The genetically identical SARS-CoV-2 infections were found in these lions, suggesting that there may be indirect transmission. All in all, the confirmation of natural SARS-CoV-2 Delta variant infections in Asian lions in India proves the urgent need for SARS-CoV-2 surveillance in wild animal species. Since lions are rare animals, they are not suitable for experimental animal models, but the death cases of large mammals may be worthy of further exploration.

## Comparison of Different Animal Models

Generally, mice are more suitable for basic research on the pathogenic mechanism of SARS-CoV-2 infection. Compared with transgenic mice, adenovirus-transduced mice do not require transgene or gene knock-in technology, which can shorten the modeling time and avoid the influence of traditional gene modification technologies. The golden hamster possesses natural susceptibility, and does not require genetic modification and adenovirus transduction, which saves labor and costs. Mice adapting to the SARS-CoV-2 strain through domestication is more economical and applicable than the above several mouse models. Rhesus monkeys have an immune system similar to that of humans, so they will be useful for testing responses to vaccines or drugs. Cynomolgus macaques and Callithrix jacchus can be used to simulate asymptomatic. Old AGM can be used to study ARDS caused by SARS-CoV-2. Non-human primates are the closest to humans, and theoretically they are the most ideal experimental animal models. However, the source of non-human primates is limited, and the costs associated with experiments are extremely high, if necessary, species similar to non-human primates can be used to substitute. Tree shrews, a class of mammals, had been shown to be close to primates in previous studies. No clinical symptoms could be observed in tree shrews after SARS-CoV-2 infection, although the virus shedding was detected from the nose, throat, anus and serum, suggesting that tree shrews may be intermediate hosts or asymptomatic carriers of SARS-CoV-2. Whether tree shrews can replace non-human primates still needs further research. Ferrets and cat can be infected with SARS-CoV-2 with no obvious clinical symptoms, and they are not conventional model organisms, but they can fill the gap in SARS-CoV-2 infection of small mammals.

## Conclusion and Perspectives

Animal infection models have been rapidly mobilized in response to COVID-19 pandemic. Although the existing experimental animals can not completely replicate human disease features, they actually provide useful tools to address the need for greater understanding of COVID-19. The selection of appropriate experimental animal models depends not only on experimental subjects, but also on practical conditions and ethical issues (Table 2). Based on published studies, genetically modified mice or adenoviruses/CRISPR-edited mice, or WT mice infected with mouse-adapted strain will undoubtedly become the most widely used animal models due to their convenience and economy, and success in replicating lung inflammation, histopathology and pneumonia of human infections. Non-human primates are closer to humans and are well suitable to simulate human respiratory virus infections because of their similar respiratory anatomy and immune response to humans compared to other animal species, which can be used to test interventions prior to human treatment. Other wild animals may be useful for studying the route of virus transmission but pose substantial logistical issues. Translation of animal experimental results to humans will enable us to clarify SARS-CoV-2 pathogenesis and develop and test preventions and interventions to combat COVID-19. With regard to the future studies, there is an urgent need for standardizing the challenge-protection model to compare different vaccines, and establishing appropriate models for evaluating the vaccine-associated enhanced respiratory disease potential.

**TABLE 2:** The main advantages and disadvantages of different animal models of SARS-CoV-2.

Animal models	Advantages	Disadvantages
Mouse	Clear genetic background Widely used in various research Humanized ACE2 receptor	Unobvious clinical symptoms No symptoms of severe illness or death hACE2 transgenic expression or viral adaptation is required
Hamster	Typical pathological features Supports high-titer replication of the virus in the lungs	No symptoms of severe illness or death Not widely used
Rhesus monkey	Close kinship with people Obvious clinical symptoms Used in viral infection research	High cost, difficult to operate No symptoms of severe illness or death More ethical issues
Cynomolgus macaques	Close kinship with people Used in viral infection research	High cost, difficult to operate No clinical symptoms (except fever) More ethical issues
African green monkey	Close kinship with people Conducive to modeling of severe cases (acute respiratory distress syndrome appears)	High cost, difficult to operate More ethical issues
Ferret	Big body, easy to be infected Respiratory tract similar to humans	The clinical symptoms are mild No weight loss or even death Low Pneumovirus Titer
Tree shrew	Close relationship with primates Lower price than primates short breeding cycle (6 weeks)	No clinical symptoms Not widely used
Cat	Low cost, easy to get Big body, easy to be infected	No clinical symptoms Not a regular model organism

## Author Contributions

LZ and YJ designed the study. LZ conducted literature search, analysis, and draft writing. SC, WZ, and HY refined the detailed research questions in the first draft. YJ and GD helped wrote the first draft and modification. All authors contributed to the article and approved the submitted version.

## Conflict of Interest

The authors declare that the research was conducted in the absence of any commercial or financial relationships that could be construed as a potential conflict of interest.

## Publisher’s Note

All claims expressed in this article are solely those of the authors and do not necessarily represent those of their affiliated organizations, or those of the publisher, the editors and the reviewers. Any product that may be evaluated in this article, or claim that may be made by its manufacturer, is not guaranteed or endorsed by the publisher.

## References

[B1] Bao L. Deng W. Huang B. Gao H. Liu J. Ren L. (2020). The pathogenicity of SARS-CoV-2 in hACE2 transgenic mice. *Nature* 583 830–833. 10.1038/s41586-020-2312-y 32380511

[B2] Blair R. V. Vaccari M. Doyle-Meyers L. A. Roy C. J. Russell-Lodrigue K. Fahlberg M. (2021). Acute respiratory distress in Aged, SARS-CoV-2-infected african green monkeys but not rhesus macaques. *Am. J. Pathol.* 191 274–282. 10.1016/j.ajpath.2020.10.016 33171111 PMC7648506

[B3] Brehm M. A. Shultz L. D. Greiner D. L. (2010). Humanized mouse models to study human diseases. *Curr. Opin. Endocrinol. Diabetes Obes* 17 120–125. 10.1097/MED.0b013e328337282f 20150806 PMC2892284

[B4] Cao B. Zhang L. Liu H. Ma S. Mi K. (2020). The dynamic expression of potential mediators of severe acute respiratory Syndrome Coronavirus 2 cellular entry in fetal, neonatal, and adult rhesus monkeys. *Front. Genet.* 11:607479. 10.3389/fgene.2020.607479 33537060 PMC7848180

[B5] Chan J. F. Zhang A. J. Yuan S. Poon V. K. Chan C. C. Lee A. C. (2020). Simulation of the clinical and pathological manifestations of coronavirus disease 2019 (COVID-19) in a golden syrian hamster model: implications for disease pathogenesis and transmissibility. *Clin. Infect. Dis.* 71 2428–2446. 32215622 10.1093/cid/ciaa325 PMC7184405

[B6] Chandrashekar A. Liu J. Martinot A. J. McMahan K. Mercado N. B. Peter L. (2020). SARS-CoV-2 infection protects against rechallenge in rhesus macaques. *Science* 369 812–817. 10.1126/science.abc4776 32434946 PMC7243369

[B7] Chen N. Zhou M. Dong X. Qu J. Gong F. Han Y. (2020). Epidemiological and clinical characteristics of 99 cases of 2019 novel coronavirus pneumonia in Wuhan, China: a descriptive study. *Lancet* 395 507–513. 10.1016/S0140-6736(20)30211-7 32007143 PMC7135076

[B8] Chu Y. K. Ali G. D. Jia F. Li Q. Kelvin D. Couch R. C. (2008). The SARS-CoV ferret model in an infection-challenge study. *Virology* 374 151–163. 10.1016/j.virol.2007.12.032 18234270 PMC2831213

[B9] Colman R. J. (2018). Non-human primates as a model for aging. *Biochim. Biophys. Acta Mol. Basis Dis.* 1864 2733–2741. 10.1016/j.bbadis.2017.07.008 28729086 PMC5772001

[B10] Deng W. Bao L. Liu J. Xiao C. Liu J. Xue J. (2020). Primary exposure to SARS-CoV-2 protects against reinfection in rhesus macaques. *Science* 369 818–823. 10.1126/science.abc5343 32616673 PMC7402625

[B11] Dinnon K. H. Leist S. R. Schäfer A. Edwards C. E. Martinez D. R. Montgomery S. A. (2020). A mouse-adapted model of SARS-CoV-2 to test COVID-19 countermeasures. *Nature* 586 560–566. 10.1038/s41586-020-2708-8 32854108 PMC8034761

[B12] Dudley J. P. Lee N. T. (2020). Disparities in age-specific morbidity and mortality from SARS-CoV-2 in China and the republic of Korea. *Clin. Infect. Dis.* 71 863–865. 10.1093/cid/ciaa354 32232322 PMC7184419

[B13] Fan Y. Huang Z. Y. Cao C. C. Chen C. S. Chen Y. X. Fan D. D. (2013). Genome of the Chinese tree shrew. *Nat. Commun.* 4:1426. 10.1038/ncomms2416 23385571

[B14] Fan Y. Ye M. S. Zhang J. Y. Xu L. Yu D. D. Gu T. L. (2019). Chromosomal level assembly and population sequencing of the Chinese tree shrew genome. *Zool. Res.* 40 506–521. 10.24272/j.issn.2095-8137.2019.063 31418539 PMC6822927

[B15] Finch C. L. Crozier I. Lee J. H. Byrum R. Cooper T. K. Liang J. (2020). Characteristic and quantifiable COVID-19-like abnormalities in CT- and PET/CT-imaged lungs of SARS-CoV-2-infected crab-eating macaques (Macaca fascicularis). *bioRxiv* [preprint]. 10.1101/2020.05.14.096727 32511338 PMC7241101

[B16] Fu L. Wang B. Yuan T. Chen X. Ao Y. Fitzpatrick T. (2020). Clinical characteristics of coronavirus disease 2019 (COVID-19) in China: a systematic review and meta-analysis. *J. Infect.* 80 656–665. 10.1016/j.jinf.2020.03.041 32283155 PMC7151416

[B17] Gao Q. Bao L. Mao H. Wang L. Xu K. Yang M. (2020). Development of an inactivated vaccine candidate for SARS-CoV-2. *Science* 369 77–81. 10.1126/science.abc1932 32376603 PMC7202686

[B18] Gaudreault N. N. Trujillo J. D. Carossino M. Meekins D. A. Morozov I. Madden D. W. (2020). SARS-CoV-2 infection, disease and transmission in domestic cats. *Emerg. Microbes Infect.* 9 2322–2332. 10.1080/22221751.2020.1833687 33028154 PMC7594869

[B19] Grein J. Ohmagari N. Shin D. Diaz G. Asperges E. Castagna A. (2020). Compassionate use of remdesivir for patients with Severe Covid-19. *N. Engl. J. Med.* 382 2327–2336. 10.1056/NEJMoa2007016 32275812 PMC7169476

[B20] Gu H. Chen Q. Yang G. He L. Fan H. Deng Y. Q. (2020). Adaptation of SARS-CoV-2 in BALB/c mice for testing vaccine efficacy. *Science* 369 1603–1607. 10.1126/science.abc4730 32732280 PMC7574913

[B21] Guan W. J. Ni Z. Y. Hu Y. Liang W. H. Ou C. Q. He J. X. (2020). Clinical characteristics of coronavirus disease 2019 in China. *N. Engl. J. Med.* 382 1708–1720. 10.1056/NEJMoa2002032 32109013 PMC7092819

[B22] Halfmann P. J. Hatta M. Chiba S. Maemura T. Fan S. Takeda M. (2020). Transmission of SARS-CoV-2 in domestic cats. *N. Engl. J. Med.* 383 592–594. 32402157 10.1056/NEJMc2013400 PMC9678187

[B23] Harcourt J. Tamin A. Lu X. Kamili S. Sakthivel S. K. Murray J. (2020). Severe acute respiratory syndrome Coronavirus 2 from patient with Coronavirus Disease, United States. *Emerg. Infect. Dis.* 26 1266–1273. 10.3201/eid2606.200516 32160149 PMC7258473

[B24] Hartman A. L. Nambulli S. McMillen C. M. White A. G. Tilston-Lunel N. L. Albe J. R. (2020). SARS-CoV-2 infection of African green monkeys results in mild respiratory disease discernible by PET/CT imaging and shedding of infectious virus from both respiratory and gastrointestinal tracts. *PLoS Pathog.* 16:e1008903. 10.1371/journal.ppat.1008903 PMC7535860 32946524

[B25] Hassan A. O. Case J. B. Winkler E. S. Thackray L. B. Kafai N. M. Bailey A. L. (2020). A SARS-CoV-2 infection model in mice demonstrates protection by neutralizing antibodies. *Cell* 182 744–753.e4. 10.1016/j.cell.2020.06.011 32553273 PMC7284254

[B26] Hu B. Huang S. Yin L. (2021). The cytokine storm and COVID-19. *J. Med. Virol.* 93 250–256. 10.1002/jmv.26232 32592501 PMC7361342

[B27] Huang C. Wang Y. Li X. Ren L. Zhao J. Hu Y. (2020). Clinical features of patients infected with 2019 novel coronavirus in Wuhan. China. *Lancet* 395 497–506. 10.1016/S0140-6736(20)30183-5 31986264 PMC7159299

[B28] Imai M. Iwatsuki-Horimoto K. Hatta M. Loeber S. Halfmann P. J. Nakajima N. (2020). Syrian hamsters as a small animal model for SARS-CoV-2 infection and countermeasure development. *Proc. Natl. Acad. Sci. U.S.A.* 117 16587–16595. 10.1073/pnas.2009799117 32571934 PMC7368255

[B29] Ishigaki H. Nakayama M. Kitagawa Y. Nguyen C. T. Hayashi K. Shiohara M. (2021). Neutralizing antibody-dependent and -independent immune responses against SARS-CoV-2 in cynomolgus macaques. *Virology* 554 97–105. 33412411 10.1016/j.virol.2020.12.013 PMC7771262

[B30] Jiang R. D. Liu M. Q. Chen Y. Shan C. Zhou Y. W. Shen X. R. (2020). Pathogenesis of SARS-CoV-2 in transgenic mice expressing human angiotensin-converting Enzyme 2. *Cell* 182 50–58.e8. 10.1016/j.cell.2020.05.027 32516571 PMC7241398

[B31] Jiao L. Li H. Xu J. Yang M. Ma C. Li J. (2021). The gastrointestinal tract is an alternative route for SARS-CoV-2 infection in a nonhuman primate model. *Gastroenterology* 160 1647–1661. 10.1053/j.gastro.2020.12.001 33307034 PMC7725054

[B32] Jin Y. H. Cai L. Cheng Z. S. Cheng H. Deng T. Fan Y. P. (2020). A rapid advice guideline for the diagnosis and treatment of 2019 novel coronavirus (2019-nCoV) infected pneumonia (standard version). *Mil. Med. Res.* 7:4. 10.1186/s40779-020-0233-6 PMC7003341 32029004

[B33] Kayesh M. E. H. Sanada T. Kohara M. Tsukiyama-Kohara K. (2021). Tree Shrew as an emerging small animal model for human viral infection: a recent overview. *Viruses* 13:1641. 10.3390/v13081641 34452505 PMC8402676

[B34] Kim G. U. Kim M. J. Ra S. H. Lee J. Bae S. Jung J. (2020). Clinical characteristics of asymptomatic and symptomatic patients with mild COVID-19. *Clin. Microbiol. Infect.* 26:948.e1-e3. 10.1016/j.cmi.2020.04.040 32360780 PMC7252018

[B35] Kim Y. I. Kim S. G. Kim S. M. Kim E. H. Park S. J. Yu K. M. (2020). Infection and rapid transmission of SARS-CoV-2 in Ferrets. *Cell Host Microbe* 27 704–709.e2. 10.1016/j.chom.2020.03.023 32259477 PMC7144857

[B36] Le Bras A. (2020). SARS-CoV-2 causes COVID-19-like disease in cynomolgus macaques. *Lab. Anim.* 49:174. 10.1038/s41684-020-0571-8 32424314

[B37] Li R. Yuan B. Xia X. Zhang S. Du Q. Yang C. (2018). Tree shrew as a new animal model to study the pathogenesis of avian influenza (H9N2) virus infection. *Emerg. Microbes Infect.* 7:166. 10.1038/s41426-018-0167-1 30301950 PMC6177411

[B38] Liu H. L. Yeh I. J. Phan N. N. Wu Y. H. Yen M. C. Hung J. H. (2020). Gene signatures of SARS-CoV/SARS-CoV-2-infected ferret lungs in short- and long-term models. *Infect. Genet. Evol.* 85:104438. 10.1016/j.meegid.2020.104438 PMC7832673 32615317

[B39] Lu C. W. Liu X. F. Jia Z. F. (2020). 2019-nCoV transmission through the ocular surface must not be ignored. *Lancet* 395:e39. 10.1016/S0140-6736(20)30313-5 32035510 PMC7133551

[B40] Lu S. Zhao Y. Yu W. Yang Y. Gao J. Wang J. (2020). Comparison of nonhuman primates identified the suitable model for COVID-19. *Signal. Transduct. Target Ther.* 5:157. 10.1038/s41392-020-00269-6 32814760 PMC7434851

[B41] Mangalmurti N. Hunter C. A. (2020). Cytokine storms: understanding COVID-19. *Immunity* 53 19–25. 10.1016/j.immuni.2020.06.017 32610079 PMC7321048

[B42] Martina B. E. Haagmans B. L. Kuiken T. Fouchier R. A. Rimmelzwaan G. F. Van Amerongen G. (2003). Virology: SARS virus infection of cats and ferrets. *Nature* 425:915. 10.1038/425915a 14586458 PMC7094990

[B43] Mishra A. Kumar N. Bhatia S. Aasdev A. Kanniappan S. Sekhar A. T. (2021). SARS-CoV-2 delta variant among asiatic lions, India. *Emerg. Infect. Dis.* 27 2723–2725. 10.3201/eid2710.211500 34545805 PMC8462327

[B44] Mohanty S. K. Satapathy A. Naidu M. M. Mukhopadhyay S. Sharma S. Barton L. M. (2020). Severe acute respiratory syndrome coronavirus-2 (SARS-CoV-2) and coronavirus disease 19 (COVID-19) - anatomic pathology perspective on current knowledge. *Diagn. Pathol.* 15:103. 10.1186/s13000-020-01017-8 32799894 PMC7427697

[B45] Pandrea I. Apetrei C. Dufour J. Dillon N. Barbercheck J. Metzger M. (2006). Simian immunodeficiency virus SIVagm.sab infection of Caribbean African green monkeys: a new model for the study of SIV pathogenesis in natural hosts. *J. Virol.* 80 4858–4867. 10.1128/JVI.80.10.4858-4867.2006 16641277 PMC1472068

[B46] Park S. J. Yu K. M. Kim Y. I. Kim S. M. Kim E. H. Kim S. G. (2020). Antiviral efficacies of FDA-approved drugs against SARS-CoV-2 Infection in Ferrets. *mBio* 11:e01114-20. 10.1128/mBio.01114-20 32444382 PMC7244896

[B47] Pruijssers A. J. George A. S. Schäfer A. Leist S. R. Gralinksi L. E. Dinnon K. H. (2020). Remdesivir inhibits SARS-CoV-2 in human lung cells and Chimeric SARS-CoV Expressing the SARS-CoV-2 RNA polymerase in mice. *Cell Rep.* 32:107940. 10.1016/j.celrep.2020.107940 PMC7340027 32668216

[B48] Roberts A. Lamirande E. W. Vogel L. Jackson J. P. Paddock C. D. Guarner J. (2008). Animal models and vaccines for SARS-CoV infection. *Virus Res.* 133 20–32. 10.1016/j.virusres.2007.03.025 17499378 PMC2323511

[B49] Roberts A. Vogel L. Guarner J. Hayes N. Murphy B. Zaki S. (2005). Severe acute respiratory syndrome coronavirus infection of golden Syrian hamsters. *J. Virol.* 79 503–511. 10.1128/JVI.79.1.503-511.2005 15596843 PMC538722

[B50] Rockx B. Kuiken T. Herfst S. Bestebroer T. Lamers M. M. Oude Munnink B. B. (2020). Comparative pathogenesis of COVID-19, MERS, and SARS in a nonhuman primate model. *Science* 368 1012–1015. 10.1126/science.abb7314 32303590 PMC7164679

[B51] See R. H. Petric M. Lawrence D. J. Mok C. P. Y. Rowe T. Zitzow L. A. (2008). Severe acute respiratory syndrome vaccine efficacy in ferrets: whole killed virus and adenovirus-vectored vaccines. *J. Gen. Virol.* 89 (Pt 9) 2136–2146. 10.1099/vir.0.2008/001891-0 18753223

[B52] Shan C. Yao Y. F. Yang X. L. Zhou Y. W. Gao G. Peng Y. (2020). Infection with novel coronavirus (SARS-CoV-2) causes pneumonia in Rhesus macaques. *Cell Res.* 30 670–677. 32636454 10.1038/s41422-020-0364-z PMC7364749

[B53] Shi D. Wu W. Wang Q. Xu K. Xie J. Wu J. (2020). Clinical characteristics and factors associated with long-term viral excretion in patients with severe acute respiratory syndrome Coronavirus 2 infection: a single-center 28-day study. *J. Infect. Dis.* 222 910–918. 10.1093/infdis/jiaa388 32614392 PMC7337834

[B54] Shi J. Wen Z. Zhong G. Yang H. Wang C. Huang B. (2020). Susceptibility of ferrets, cats, dogs, and other domesticated animals to SARS-coronavirus 2. *Science* 368 1016–1020. 10.1126/science.abb7015 32269068 PMC7164390

[B55] Sims A. C. Baric R. S. Yount B. Burkett S. E. Collins P. L. Pickles R. J. (2005). Severe acute respiratory syndrome coronavirus infection of human ciliated airway epithelia: role of ciliated cells in viral spread in the conducting airways of the lungs. *J. Virol.* 79 15511–15524. 16306622 10.1128/JVI.79.24.15511-15524.2005 PMC1316022

[B56] Struyf T. Deeks J. J. Dinnes J. Takwoingi Y. Davenport C. Leeflang M. M. (2020). Signs and symptoms to determine if a patient presenting in primary care or hospital outpatient settings has COVID-19 disease. *Cochrane Database Syst. Rev.* 7:Cd013665. 10.1002/14651858.CD013665 32633856 PMC7386785

[B57] Sun J. Zhuang Z. Zheng J. Li K. Wong R. L. Liu D. (2020). Generation of a broadly useful model for COVID-19 pathogenesis, vaccination, and treatment. *Cell* 182 734–743.e5. 32643603 10.1016/j.cell.2020.06.010 PMC7284240

[B58] Sun S. H. Chen Q. Gu H. J. Yang G. Wang Y. X. Huang X. Y. (2020). A mouse model of SARS-CoV-2 infection and pathogenesis. *Cell Host Microbe* 28 124–133.e4. 10.1016/j.chom.2020.05.020 32485164 PMC7250783

[B59] Takayama K. (2020). In Vitro and animal models for SARS-CoV-2 research. *Trends Pharmacol. Sci.* 41 513–517. 32553545 10.1016/j.tips.2020.05.005 PMC7260555

[B60] van Doremalen N. Lambe T. Spencer A. Belij-Rammerstorfer S. Purushotham J. N. Port J. R. (2020). ChAdOx1 nCoV-19 vaccine prevents SARS-CoV-2 pneumonia in rhesus macaques. *Nature* 586 578–582. 10.1038/s41586-020-2608-y 32731258 PMC8436420

[B61] Walsh N. C. Kenney L. L. Jangalwe S. Aryee K. E. Greiner D. L. Brehm M. A. (2017). Humanized mouse models of clinical disease. *Annu. Rev. Pathol.* 12 187–215. 27959627 10.1146/annurev-pathol-052016-100332 PMC5280554

[B62] Wan Y. Shang J. Graham R. Baric R. S. Li F. (2020). Receptor recognition by the novel Coronavirus from Wuhan: an analysis based on decade-long structural studies of SARS Coronavirus. *J. Virol.* 94:e127-20. 10.1128/JVI.00127-20 31996437 PMC7081895

[B63] Wang H. Zhang Y. Huang B. Deng W. Quan Y. Wang W. (2020). Development of an inactivated vaccine candidate, BBIBP-CorV, with potent protection against SARS-CoV-2. *Cell* 182 713–721.e9. 10.1016/j.cell.2020.06.008 32778225 PMC7275151

[B64] WHO (2021). WHO Coronavirus (COVID-19) Dashboard | WHO Coronavirus (COVID-19) Dashboard With Vaccination Data. Available Online at https://covid19.who.int/ (Accessed October 1, 2021).

[B65] Woolsey C. Borisevich V. Prasad A. N. Agans K. N. Deer D. J. Dobias N. S. (2021). Establishment of an African green monkey model for COVID-19 and protection against re-infection. *Nat. Immunol.* 22 86–98. 33235385 10.1038/s41590-020-00835-8 PMC7790436

[B66] Xu L. Yu D. D. Ma Y. H. Yao Y. L. Luo R. H. Feng X. L. (2020). COVID-19-like symptoms observed in Chinese tree shrews infected with SARS-CoV-2. *Zool. Res.* 41 517–526. 10.24272/j.issn.2095-8137.2020.053 32701249 PMC7475013

[B67] Xu Z. Shi L. Wang Y. Zhang J. Huang L. Zhang C. (2020). Pathological findings of COVID-19 associated with acute respiratory distress syndrome. *Lancet Respir. Med.* 8 420–422. 32085846 10.1016/S2213-2600(20)30076-X PMC7164771

[B68] Yang X. Yu Y. Xu J. Shu H. Xia J. Liu H. (2020). Clinical course and outcomes of critically ill patients with SARS-CoV-2 pneumonia in Wuhan, China: a single-centered, retrospective, observational study. *Lancet Respir. Med.* 8 475–481. 10.1016/S2213-2600(20)30079-5 32105632 PMC7102538

[B69] Yu J. Tostanoski L. H. Peter L. Mercado N. B. McMahan K. Mahrokhian S. H. (2020). DNA vaccine protection against SARS-CoV-2 in rhesus macaques. *Science* 369 806–811. 32434945 10.1126/science.abc6284 PMC7243363

[B70] Yu P. Qi F. Xu Y. Li F. Liu P. Liu J. (2020). Age-related rhesus macaque models of COVID-19. *Animal Model Exp. Med.* 3 93–97. 10.1002/ame2.12108 32318665 PMC7167234

[B71] Zhang L. Shen Z. L. Feng Y. Li D. Q. Zhang N. N. Deng Y. Q. (2019). Infectivity of Zika virus on primary cells support tree shrew as animal model. *Emerg. Microbes Infect.* 8 232–241. 30866776 10.1080/22221751.2018.1559707 PMC6455147

[B72] Zhang X. Cai H. Hu J. Lian J. Gu J. Zhang S. (2020). Epidemiological, clinical characteristics of cases of SARS-CoV-2 infection with abnormal imaging findings. *Int. J. Infect. Dis.* 94 81–87. 10.1016/j.ijid.2020.03.040 32205284 PMC7270493

[B73] Zhao Y. Wang J. Kuang D. Xu J. Yang M. Ma C. (2020). Susceptibility of tree shrew to SARS-CoV-2 infection. *Sci. Rep.* 10:16007. 10.1038/s41598-020-72563-w PMC7525503 32994418

[B74] Zhou P. Yang X. L. Wang X. G. Hu B. Zhang L. Zhang W. (2020). A pneumonia outbreak associated with a new coronavirus of probable bat origin. *Nature* 579 270–273. 10.1038/s41586-020-2012-7 32015507 PMC7095418

[B75] Zhou Z. Zhang M. Wang Y. Zheng F. Huang Y. Huang K. (2020). Clinical characteristics of older and younger patients infected with SARS-CoV-2. *Aging* 12 11296–11305. 10.18632/aging.103535 32575073 PMC7343458

[B76] Zhu N. Zhang D. Wang W. Li X. Yang B. Song J. (2020). A Novel coronavirus from patients with Pneumonia in China, 2019. *N. Engl. J. Med.* 382 727–733. 31978945 10.1056/NEJMoa2001017 PMC7092803

